# The major plant sphingolipid long chain base phytosphingosine inhibits growth of bacterial and fungal plant pathogens

**DOI:** 10.1038/s41598-022-05083-4

**Published:** 2022-01-20

**Authors:** René Glenz, Agnes Kaiping, Delia Göpfert, Hannah Weber, Benjamin Lambour, Marvin Sylvester, Christian Fröschel, Martin J. Mueller, Mohamed Osman, Frank Waller

**Affiliations:** Julius-von-Sachs Institute of Biosciences, Pharmaceutical Biology, Julius-Maximilians-Universität Würzburg, Julius-von-Sachs-Platz 2, 97082 Würzburg, Germany

**Keywords:** Antimicrobials, Plant immunity, Microbe, Plant breeding

## Abstract

Sphingolipid long chain bases (LCBs) are building blocks of sphingolipids and can serve as signalling molecules, but also have antimicrobial activity and were effective in reducing growth of a range of human pathogens. In plants, LCBs are linked to cell death processes and the regulation of defence reactions against pathogens, but their role in directly influencing growth of plant-interacting microorganisms has received little attention. Therefore, we tested the major plant LCB phytosphingosine in in vitro tests with the plant pathogenic fungi *Verticillium longisporum*, *Fusarium graminearum* and *Sclerotinia sclerotiorum*, the plant symbiotic fungal endophyte *Serendipita indica*, the bacterial pathogens *Pseudomonas syringae* pv. *tomato* (*Pst*), *Agrobacterium tumefaciens*, and the related beneficial strain *Rhizobium radiobacter*. Phytosphingosine inhibited growth of these organisms at micromolar concentrations. Among the fungal pathogens, *S. sclerotiorum* was the most, and *F. graminearum* was the least sensitive. 15.9 μg/mL phytosphingosine effectively killed 95% of the three bacterial species. Plant disease symptoms and growth of *Pst* were also inhibited by phytosphingosine when co-infiltrated into Arabidopsis leaves, with no visible negative effect on host tissue. Taken together, we demonstrate that the plant LCB phytosphingosine inhibits growth of plant-interacting microorganisms. We discuss the potential of elevated LCB levels to enhance plant pathogen resistance.

## Introduction

Sphingolipid long-chain bases (LCBs) are key components required for the formation of sphingolipids and are also intracellular signalling molecules. Sphingolipids occur in cellular membranes of all eukaryotes, but only a few bacterial genera^1–3^. LCBs are comprised of an amino alcohol with typically 18 carbon atoms in plants, characterized by hydroxyl groups at C1 and C3 positions (d18:0; dihydrosphinganine), or an additional hydroxyl group at C4 (t18:0; phytosphingosine), and an amine group at C2. Sphingolipids are formed by linking this amine group to a fatty acid (with chain lengths between 14 and 26 carbon atoms), and further modifications, e.g. sugar residues and/or phosphate groups linked to the C1 position. Beside their role for sphingolipid biosynthesis, LCBs can function as intra- and intercellular signal transduction molecules. In model systems like yeast, as well as in humans, LCBs regulate cellular processes such as differentiation, cell division, inflammation and cell death^4,5^.

In plants, LCBs are implicated in regulating stomatal closure^6,7^, and elevated levels of LCBs are closely correlated with cell death processes. LCB treatment induces cell death^8–10^, which could also be caused by a block of sphingolipid biosynthesis, leading to increased cellular levels of LCBs^8,11,12^. Programmed cell death in *Arabidopsis thaliana* induced by recognition of an avirulence protein of the bacterial pathogen *Pseudomonas syringae* pv. *tomato* (*Pst*) leads to elevated levels of the LCB phytosphingosine^9^. A role for LCBs in plant pathogen defence is also supported by genetic evidence, e.g. in an Arabidopsis mutant with a disturbed LCB turnover^13^. Taken together, these results suggest that high levels of plant LCBs can be positively correlated with enhanced defence responses resulting in reduced pathogen growth after infection.

We hypothesized that elevated LCB levels in plants could also lead to a direct inhibition of pathogen growth. Antimicrobial activity of LCBs was shown for a range of gram-positive and negative bacteria, among them human pathogens (such as *Pseudomonas aeruginosa* or *Neisseria meningitidis*) and *Escherichia coli* strains, but also fungal species such as *Candida albicans*^14–18^. This activity was shown for several LCB species, including those LCBs mainly present in plants, phytosphingosine (t18:0) and dihydrosphingosine (d18:0)^19^. To our knowledge, antimicrobial effects of LCBs against plant pathogens received attention only recently^20^, and have not been systematically assessed. Therefore, we aimed to test the antimicrobial effect of phytosphingosine on those bacterial and fungal species interacting with plants.

We choose four well-studied fungal species with different lifestyles: the ascomycete species *Fusarium graminearum*, a plant pathogen causing wheat head blight^21,22^, *Sclerotinia sclerotiorum*, causing stem rot^23,24^, the vascular pathogen *Verticillium longisporum,* causing wilt, e.g. in canola^25,26^, and the beneficial root-colonising basidiomycete *Serendipita indica*^27–29^. Three different plant-interacting bacteria were also included: the leaf model pathogen *Pseudomonas syringae* pv. *tomato*, a strain of *Agrobacterium tumefaciens,* and the related non-pathogenic strain *Rhizobium radiobacter* F4^30,31^. We analysed the growth-inhibiting effect of phytosphingosine on these fungal and bacterial species in vitro. For the bacterial model pathogen *Pst* we also tested the effect of phytosphingosine on bacterial growth and host tissue damage in leaves of *Arabidopsis thaliana* using co-infiltration experiments.

## Results

### Phytosphingosine inhibits mycelium growth of plant-interacting fungi in vitro

**Figure 1:**
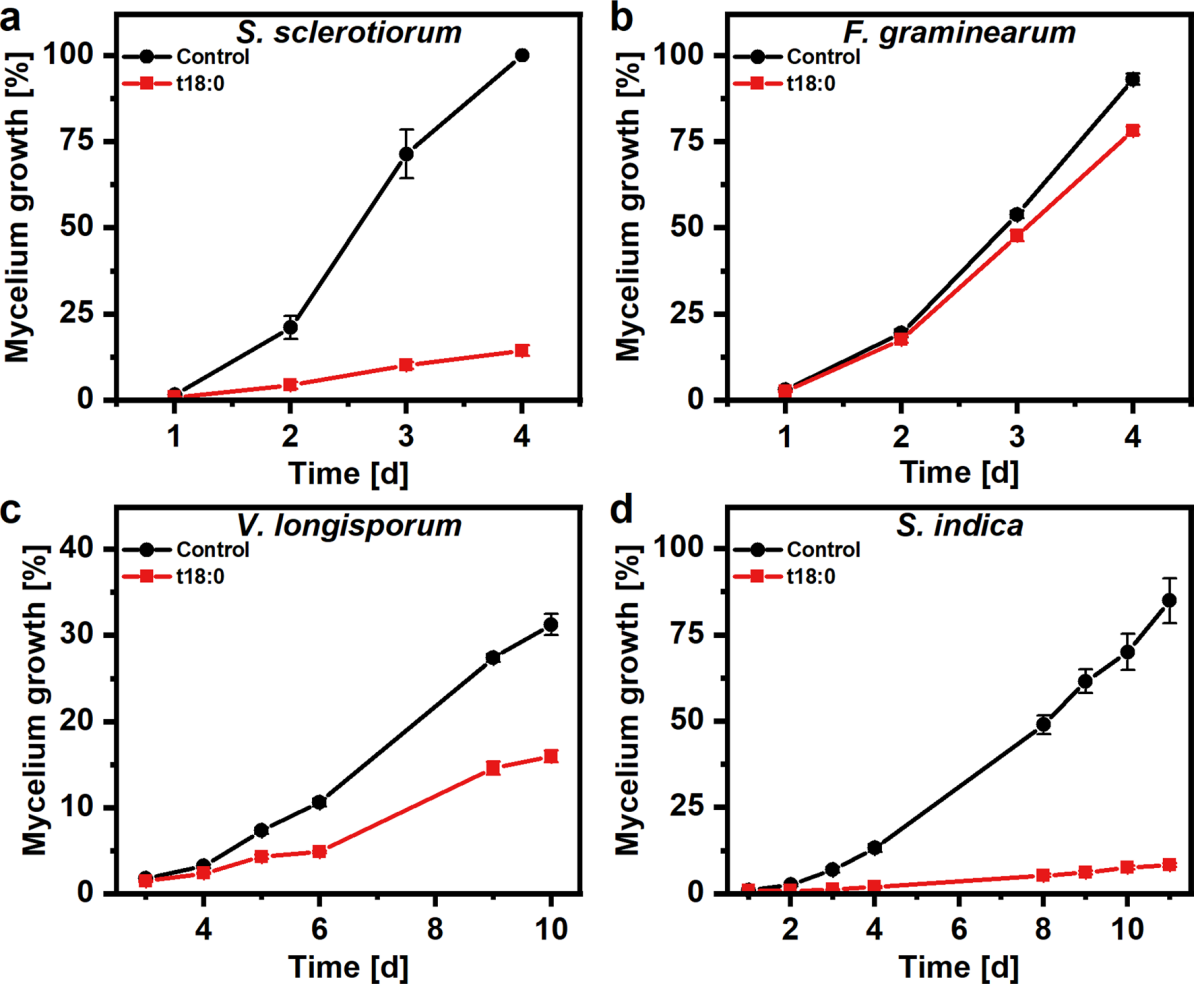
Phytosphingosine inhibits growth of fungal hyphae on agar medium. (**a**) *Sclerotinia sclerotiorum*, (**b**) *Fusarium graminearum*, (**c**) *Verticillium longisporum* and (**d**) *Serendipita indica*. Agar plates contained different media optimal for each fungus (see “Material and methods”). Media were supplemented with either 80 µM phytosphingosine (t18:0) or were prepared with solvent (1% DMSO; Control) only. Plates were inoculated in the center with a mycelial plug (**a**,**b**,**d**) or 10 µL of spore solution (containing 5000 spores) (**c**), and growth of mycelia on the surface of the medium was documented over time. Mycelial growth is presented as the percentage of the total area of the plate covered by the mycelium. Values are means of 9–15 plates, with error bars indicating standard error of the mean (SEM).

We tested *Sclerotinia sclerotiorum*, *Fusarium graminearum*, *Verticillium longisporum* and *Serendipita indica* for their sensitivity to phytosphingosine (t18:0). Respective agar media (see material and methods) contained 80 μM t18:0 or the solvent (1% DMSO) as control. Plates were inoculated in the centre and growth of mycelia on the surface of the medium was documented for 10–11 days or until the full plate was covered with mycelium (Suppl. Fig. 1). Mycelial growth was determined as the percentage of the total area of the plate covered by the mycelium. All fungi were negatively affected in mycelial growth by the presence of t18:0, indicated by analysis of variance (ANOVA; p < 0.001) for the effect of t18:0, with the factor ‘time’ as a covariate. The mycelial area was reduced by phytosphingosine by 90% (*S. indica*), 85% (*S. sclerotiorum*), 49% (*V. longisporum*) and 16% (*F. graminearum*) at the end of the observation period (Fig. 1). Beside the reduced mycelial plate coverage, fungal structures on the plate were altered, forming a more compact mycelial network in the presence of t18:0 (Suppl. Fig. 1).

### Phytosphingosine inhibits biomass increase of plant-interacting fungi in vitro

**Figure 2:**
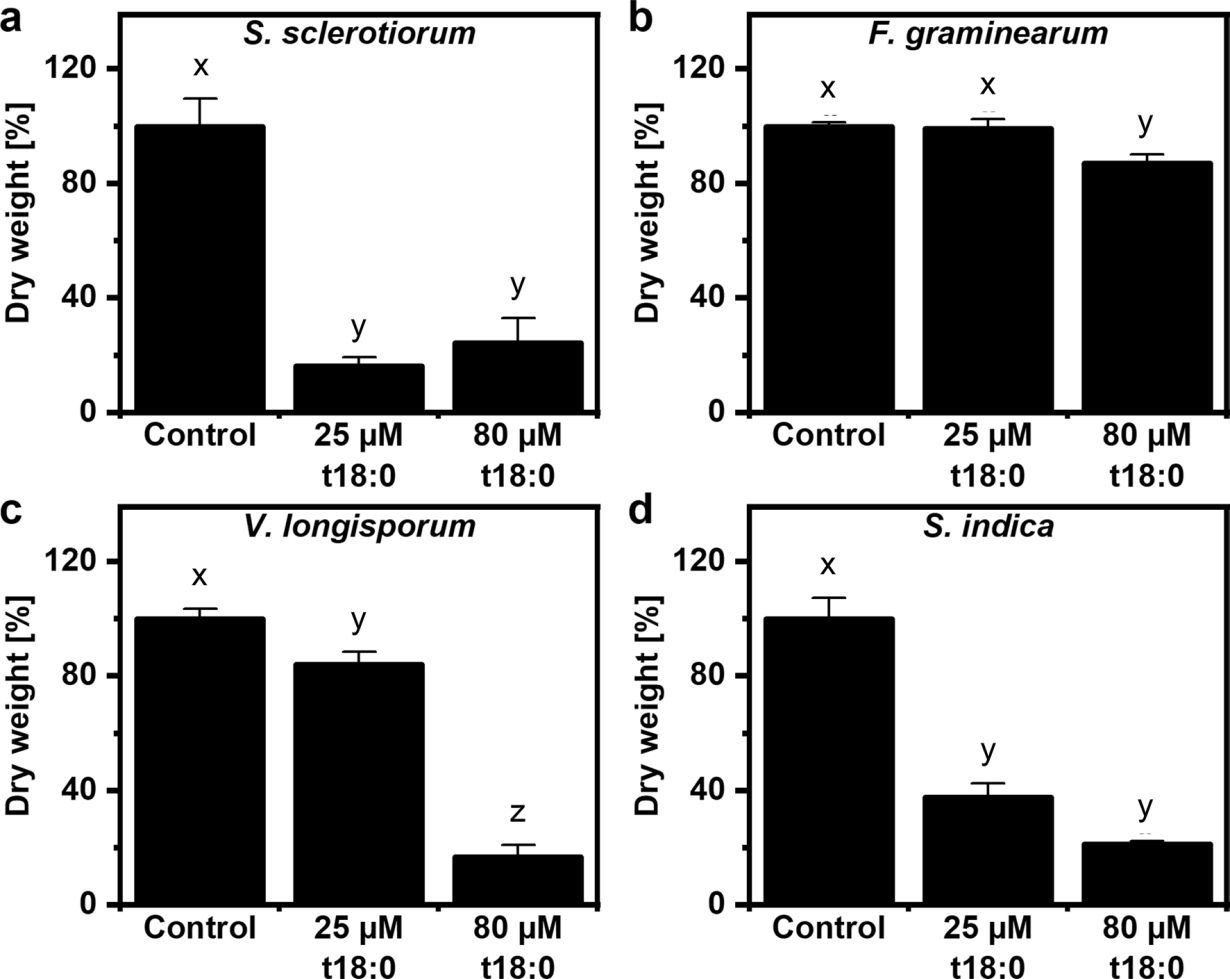
Phytosphingosine inhibits fungal growth in liquid medium. (**a**) *Sclerotinia sclerotiorum*, (**b**) *Fusarium graminearum*, (**c**) *Verticillium longisporum* and (**d**) *Serendipita indica*. Erlenmeyer flasks with 20 mL of medium were inoculated with a mycelial plug (**a**,**b**,**d**) or 10 µL of spore solution (containing 5000 spores) (**c**). The respective media either contained the indicated concentrations of phytosphingosine (t18:0), or solvent (1% DMSO; Control) only. Fungal dry weight was determined after several days of growth (**a**,**b**: 5 days, **c**: 10 days, **d**: 12 days) by collecting and drying fungal mycelia on filter paper. Dry weight is expressed as percentage of the control treatment which was set to 100%. Values are means of four to eight flasks, with error bars indicating standard error of the mean (SEM). Different letters within each diagram indicate statistical differences according to Tukey-HSD (p < 0.05) after one-way ANOVA.

In addition to plate experiments, we also tested if fungal biomass production was affected by t18:0. The four fungi were grown in appropriate liquid media with and without phytosphingosine. After several days of growth, fungal structures were harvested before reaching the stationary phase (Fig. 2). Using 80 μM t18:0, a significant reduction of fungal dry weight by 20 to 80% could be observed for all fungal species. Again, *S. sclerotiorum* and *S. indica* were affected mostly by the presence of the LCB, while the effect on *F. graminearum* was smaller. We also tested a lower concentration, 25 μM t18:0, which had no effect on *F. graminearum*, but was able to reduce biomass production by 84% for *S. sclerotiorum*, by 62% for *S. indica* and 16% for *V. longisporum* (Fig. 2).

### Phytosphingosine kills different plant-interacting bacteria

To determine the sensitivity of plant-interacting bacteria towards phytosphingosine, we performed a killing assay for the plant-interacting bacterial species *Pseudomonas syringae* pv. *tomato*, *Agrobacterium tumefaciens* and *Rhizobium radiobacter *F4. Bacteria with an initial OD of 0.001 were inoculated in different concentrations of t18:0 for 15–20 min. Bacterial survival was determined by plating serial dilutions and counting colony numbers. Killing efficiency was expressed as the percentage of colonies formed relative to the control conditions with solvent alone (Suppl. Fig. 2). 7.9 μg/ml and 15.9 μg/ml t18:0 (corresponding to concentrations of 25 and 50 μM) were sufficient to reduce bacterial survival by more than 50% or 95%, respectively, for the three bacterial species.

### Phytosphingosine, not phytosphingosine-phosphate, inhibits growth of *Pseudomonas syringae*

**Figure 3:**
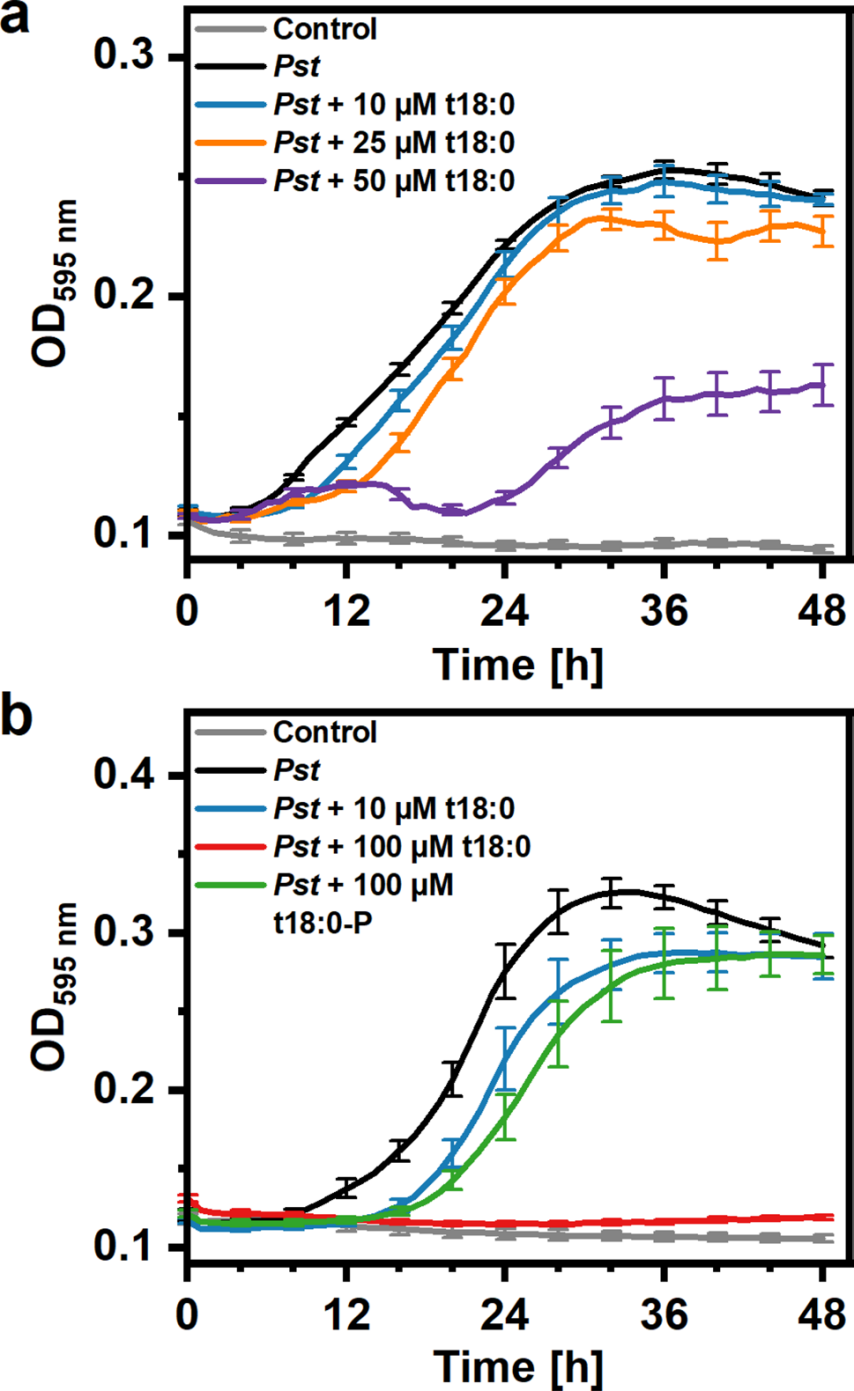
Phytosphingosine inhibits *Pseudomonas syringae* growth in liquid medium. Wells from cell culture plates with 0.2 mL of KB medium were inoculated with *Pseudomonas syringae* pv. *tomato* (*Pst*) DC3000 suspension (OD = 0.01) or a solvent control (2% DMSO in 10 mM MgCl_2_) without bacteria. The medium either contained the indicated concentrations of phytosphingosine (t18:0), phytosphingosine-1-phosphate (t18:0-P), or solvent (2% DMSO) only. Plates were incubated under constant shaking at 28 °C for the indicated time in a plate reader. OD at 595 nm was measured each hour and values are means of 11 (**a**) or 8 (**b**) wells. Error bars indicating standard error of the mean (SEM) are depicted for values every four hours for better visualization.

As MIC values were similar for the three bacterial species, further tests were performed only with the widely used model plant pathogen *Pseudomonas syringae* pv. *tomato*. In addition to the plate experiments, we wanted to test the effect of t18:0 on bacterial growth over time. For this purpose, we continuously measured the optical density (OD) after inoculation of King’s B (KB) medium with an OD of 0.01 (corresponding to 5 × 10^6^ culture forming units (cfu) / mL bacteria). Under these conditions (with a higher initial concentration of bacteria, as compared with MIC tests), 50 μM t18:0 was required to substantially reduce growth over time (Fig. 3 a), with Tukeys *post-hoc* test showing significant differences (p < 0.05), e.g. at 12, 24, 36 and 48 h. Addition of 100 μM t18:0 completely abolished bacterial growth for 48 h (p < 0.05 in a Tukey *post-hoc* test at 12, 24, 36, 48 h), while the same concentration of the phosphorylated form of t18:0, t18:0-P, was not able to reduce bacterial growth (Fig. 3b).

### Phytosphingosine reduces disease symptoms and inhibits growth of *Pseudomonas syringae* when co-infiltrated into Arabidopsis leaves

**Figure 4:**
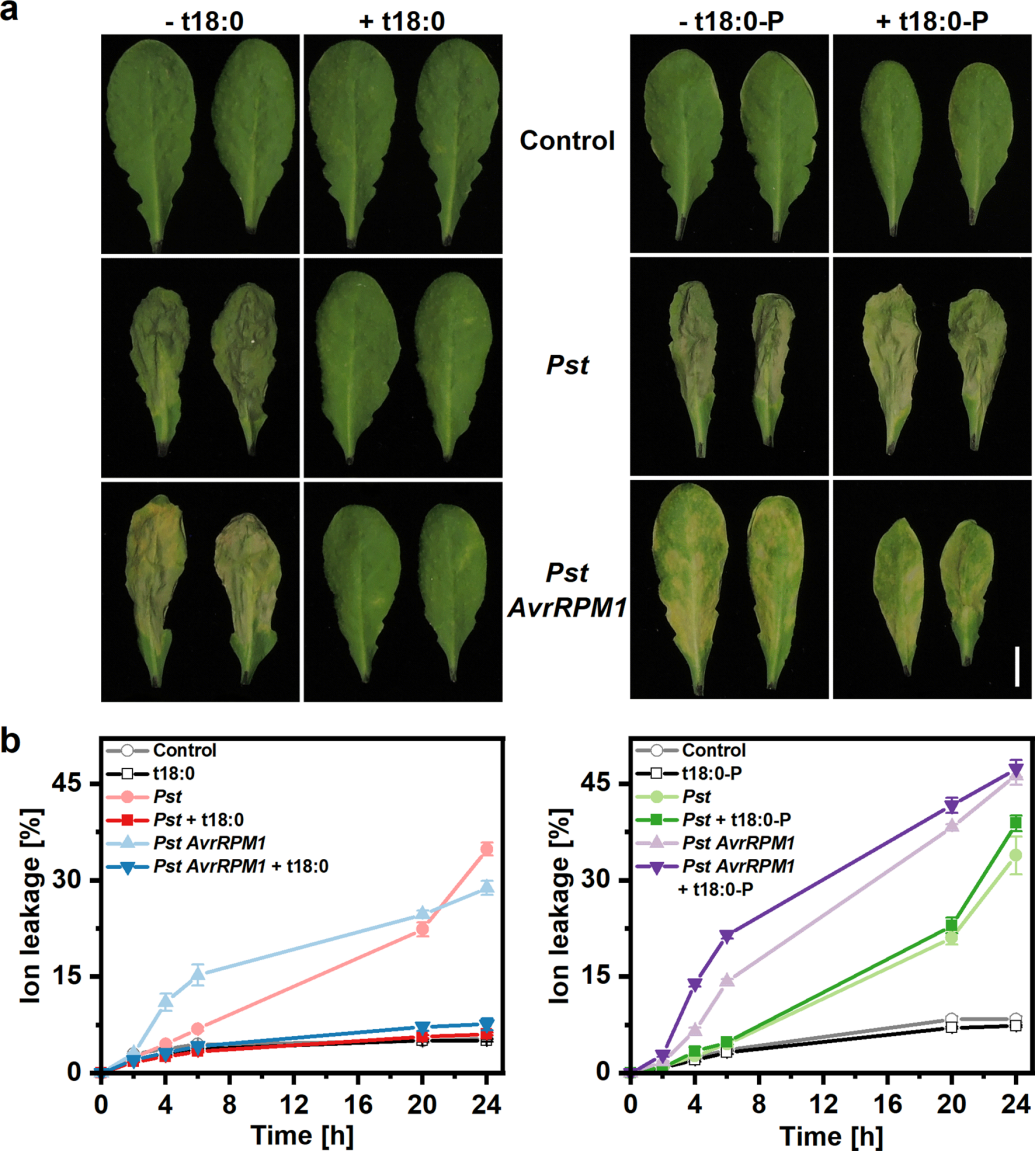
Phytosphingosine co-infiltration with *Pseudomonas syringae* reduces disease symptoms in Arabidopsis leaves. Two strains of *Pseudomonas syringae* pv. *tomato (Pst)*, *Pst* DC3000 (virulent) and *Pst* DC3000 *AvrRPM1* (avirulent) were diluted to OD 0.01 in either 10 mM MgCl_2_ containing 100 µM phytosphingosine (t18:0), 100 µM phytosphingosine-1-phosphate (t18:0-P), or solvent (2% DMSO; Control) only. Leaves were infiltrated and photographs were taken 72 h after infiltration (**a**) to assess leaf damage. Scale bar represents 1 cm. To quantify the development of host cell death (**b**), infiltrated leaves were subjected to leaf disc ion leakage assays. Leaf discs were floated on ultrapure H_2_O and conductivity of the solution was determined for 24 h. Cell death was measured as the conductivity of the solution at respective time points relative to the conductivity of the boiled sample at the end of the experiment, which was set to 100%. The time points indicated represent the time after immersion of leaf discs in ultrapure water. Values are means of three independent samples, each consisting of five leaf discs with error bars indicating standard error of the mean (SEM).

In addition to in vitro experiments, we tested the ability of t18:0 to inhibit growth of *Pseudomonas syringae in planta*. We used a well-established method for co-infiltration^13^ and infiltrated in separate experiments two different strains of *Pst* into leaves of *Arabidopsis thaliana*. Infiltration of the virulent strain *Pst* DC3000 leads to wilting symptoms of the leaves 24–48 h after infiltration, depending on the amount of bacteria used for inoculation. The strain *Pst AvrRPM1* (expressing the avirulence determinant avrRpm1) induces the hypersensitive response^32^, a programmed cell death reaction of the host plant, visible as yellow lesions (Fig. 4). When t18:0 was co-infiltrated with the bacteria, no disease symptoms could be observed (Fig. 4). Co-infiltration of t18:0-P was not effective in reducing disease symptoms (Fig. 4).

We also quantified cell death reactions after *Pst* co-infiltration with t18:0 or t18:0-P in leaves using ion leakage measurements of leaf discs derived from infiltrated leaves. Cell death induction by infiltration with bacteria was abolished when *Pst* was co-infiltrated with t18:0 (ANOVA p < 0.0001) in both bacterial strains. t18:0-P had no significant growth-reducing effect (ANOVA p = 0.264) for *Pst*, and only a slight and transient positive effect (ANOVA p = 0.003) for *Pst AvrRPM1* (Fig. 4b).

To determine, if the observed effects of t18:0 co-infiltration on visible disease symptoms and host cell death were due to reduced growth of *Pst* bacteria in the leaf, we quantified the amount of *Pst* DC3000 present in the leaves 1, 24 and 48 h post infiltration with and without t18:0. Relative bacterial quantification was performed by extracting DNA from leaf samples, and subsequent quantitative PCR (qPCR) using a *Pseudomonas*-specific and a plant-specific primer pair. The ratio of bacterial DNA per plant DNA increased between 10 and 30-fold from 1 to 24 h after infiltration when no t18:0 was co-infiltrated. Presence of 100 μM t18:0 prevented an increase of bacterial DNA per plant DNA 24 and 48 h after infiltration (Supplementary Fig. 3), indicating t18:0-dependent growth inhibition of *Pst* in the leaf.

## Discussion

Plant pathogens have received little attention with respect to their susceptibility to LCBs, although plants can upregulate phytosphingosine levels after pathogen recognition during the hypersensitive response^9^, and a range of LCB species, including phytosphingosine, are able to inhibit growth of *E. coli* and a range of human pathogens^19^. Our initial experiments revealed that t18:0 could inhibit growth of four plant-interacting fungal species with diverse life styles. Mycelium growth was visibly inhibited on agar media containing 80 μM phytosphingosine (Fig. 1), and hyphal structures appeared more compact in the presence of t18:0 (Suppl. Fig. 1). Fungal growth was also significantly reduced by phytosphingosine in terms of biomass production in liquid medium (Fig. 2), with growth reduction up to 5.5-fold. Among the fungi tested, *S. indica* and *S. sclerotiorum* were most sensitive, with 25 μM t18:0 being sufficient to reduce biomass by 62 and 83%, respectively. Published minimum fungicidal concentrations of t18:0 differ, depending on the fungal species and strain, as well as media and growth conditions used. Our results are in a similar range as minimum fungicidal concentrations reported for the unicellular fungus *Saccharomyces cerevisiae* (> 16 μM)^33^. Published values for *Candida albicans* varied between 3.2 μM^34^, and 10 to 100 μM t18:0 required for a 50% growth reduction^17^.

*F. graminearum* was the least sensitive fungal species (Fig. 1, 2, Suppl. Fig. 1). We do not know yet why these differences in sensitivity exist. *Fusarium* ssp. may have an intrinsic higher capacity to cope with high LCB levels, as some *Fusarium* species (but not *F. graminearum*) produce the toxin Fumonisin B1, a virulence factor increasing LCB levels in host cells by blocking ceramide synthase activity^2,35^. In general, sensitivity of fungi to externally applied phytosphingosine may depend on the uptake and on the capacity to degrade or incorporate this LCB into sphingolipids. LCBs are readily taken up via the fungal plasma membrane, as shown for d18:0 and t18:0 in *Aspergillus nidulans*^36^. An insertion of LCBs into the membrane, thereby compromising membrane integrity or functioning is also possible, as observed in *C. albicans*^17^. Also, high LCB levels can induce the endoplasmic reticulum stress surveillance pathway^37^, or can trigger mitochondrial reactive oxygen species-mediated damage^38^. As LCBs can also be intracellular signalling molecules in fungi, which can trigger cell death in *Candida* and *A. nidulans*^34,36^, it is likely that multiple cellular processes contribute to LCB-mediated growth inhibition and toxicity.

Phytosphingosine affected all three plant-interacting bacteria, *Pseudomonas syringae* pv. *tomato (Pst)*, *Agrobacterium tumefaciens*, and *Rhizobium radiobacter.* In the three species, 25 μM (7.9 μg/ml) t18:0 was sufficient to kill 50% of bacteria after 15–20 min incubation. Sensitivity of these plant-interacting bacteria was therefore in a similar range as MIC values reported for *E. coli* and *Staphylococcus aureus* (3.9 and 1.6 μg/ml)^19^. The antibacterial effect seems to require the specific molecular structure of t18:0, as treatments of *Pst* with t18:0-P (t18:0 with an additional phosphate group) had no significant effect on bacterial growth (Figs. 3, 4). It is also possible that differences in solubility and membrane permeability (e.g. due to the phosphate group) are responsible for this difference. The antibacterial effects of LCBs might result from membrane damage, as LCBs rapidly associate with treated *E. coli* and *S. aureus* and cause ultrastructural damage^39^. Functional tests with *P. aeruginosa* and *S. aureus* show that sphingosine binding to the bacterial membrane lipid cardiolipin was responsible for its antibacterial effect, possibly due to re-arrangement of cardiolipin molecules in the bacterial membrane leading to its permeabilisation^40^. Cardiolipin is also present in *Pst*^41^.

To determine if phytosphingosine can also inhibit bacterial growth in the leaf, we co-infiltrated *Pseudomonas syringae* with t18:0. *Pst* enters intact leaves via the stomata, and initial growth takes place in the apoplastic space. We mimicked this situation by infiltrating a bacteria-LCB mixture via the stomata into the apoplastic space of Arabidopsis leaves, a well-established method to infect Arabidopsis^13,42^. Presence of 100 μM t18:0 clearly reduced disease symptoms and host tissue damage was reduced when t18:0 was present, while t18:0-P was not effective (Fig. 4). Quantification of *Pseudomonas* bacteria in co-infiltrated leaves 24 h and 48 h after infiltration confirmed that t18:0 co-infiltration inhibited growth of *Pst* within the plant leaf (Suppl. Fig. 3). In line with our results for t18:0, published co-infiltration experiments in Arabidopsis with dihydrosphingosine (d18:0) showed strongly reduced host cell death symptoms with *Pst* strain DC3000, and similar, but weaker, effects on *Pst AvrRPM1*^13^. Co-infiltration with t18:0-P resulted in reduced host cell death^13^, which we did not observe (Fig. 4). We currently do not know the reason for this discrepancy. Magnin-Robert et al. (2015) discuss the effects of d18:0 and t18:0-P on *Pst* in the leaf as resulting from the influence of LCBs on host cells, thereby influencing pathogen spread in the host tissue. Here, we provide evidence that t18:0 can have a direct growth-inhibiting effect on *Pseudomonas syringae*, both in vitro and *in planta*.

The antimicrobial activity of t18:0 shown here raises the question if increased LCB concentrations in the leaf may be a mechanism to protect plants against pathogens like *Pst*. Our co-infiltration results, as well as results from^13^, show that LCB concentrations sufficient to stop *Pst* growth were not detrimental to host tissue integrity. Therefore, production and possibly the export into the apoplast of t18:0 could be a mechanism to restrict pathogen growth in the leaf. In total extracts of *Pst*-infected Arabidopsis leaves, t18:0 levels were around 25 nmol/g dry weight^1^. These levels would correspond to a concentration of 2.5 μM on a fresh weight basis, considerably lower than effective concentrations of 25 μM determined here. However, t18:0 is not uniformely distributed in the leaf, and export of t18:0 in vesicles out of the cells into the apoplast could result in much higher concentrations at the interface of the plant cell walls with the bacteria, and result in effective killing of *Pseudomonas syringae*.

Recently, t18:0 was detected in wheat root exudates, raising the fascinating possibility that plants indeed may employ phytosphingosine export into the apoplastic space to influence microbial growth^20^. In light of these results, increases in t18:0 levels measured in the leaf after *Pst* recognition^9^ could be sufficient to inhibit bacterial growth, if this antimicrobial compound is localized at the apoplastic cellular surfaces in contact with the bacteria.

Phytosphingosine also inhibited the potentially beneficial microorganisms *S. indica* and *Rhizobiom radiobacter* F4, and phytosphingosine soil treatment was shown to affect the microbial community composition of the watermelon rhizosphere^20^. Approaches to manipulate plant t18:0 levels for enhanced pathogen resistance would have to take this into account, e.g. by constructing transgenic plants able to export t18:0 into the apoplast under the control of a highly specific pathogen-inducible promotor.

## Methods

### Organisms and media used

*Arabidopsis thaliana* (thale cress; NCBI:txid3702) plants were ecotype *Col-*0 (Nottingham Arabidopsis Stock Center N1092). Plants were grown on soil in a growth chamber under a 9 h/15 h short day cycle at 22/20 °C (70% humidity). *Pseudomonas syringae* pv*. tomato* DC3000 (*Pst*) strains with/without *AvrRPM1* were used^42,43^. *Pseudomonas syringae* was initially cultured from glycerol stock solutions in King`s B (KB) medium with 50 μg/ml rifampicin and, for the *AVRRpm1* strain, with 5 μg/ml tetracycline, at 28 °C. Bacterial suspension cultures were grown over night, subcultured in fresh medium and cells were collected during the exponential growth phase by centrifugation. Bacteria were then washed and resuspended in 10 mM MgCl_2_ to the desired optical density. *Agrobacterium tumefaciens* GV3101^44^ was cultured in YEB medium supplemented with gentamycin (25 μg/ml) and rifampicin (10 μg/ml). *Rhizobium radiobacter*^31^ was provided by Prof. K.H. Kogel, Giessen University, Germany, and cultured in LB medium supplemented with gentamycin (75 μg/ml). *Serendipita indica / Piriformospora indica* isolate DSM11827 was obtained from the DSMZ (Deutsche Sammlung von Mikroorganismen und Zellkulturen GmbH, Braunschweig, Germany). *Fusarium graminearum* was provided by Prof. R. Hückelhoven, Technical University Munich, Germany. *Verticillium longisporum* strain *Vl*43^26^ was obtained from Prof. W. Dröge-Laser, Würzburg University, Germany. *Sclerotinia sclerotiorum* was obtained from Dr. H. Stotz^23^. Plant experiments were performed in accordance with national/institutional guidelines and regulations.

### Preparation of compounds for antimicrobial activity tests

Phytosphingosine (t18:0) and phytosphingosine-phosphate (t18:0-P) (both with > 99% purity) were purchased from Avanti Polar Lipids (Alabaster, USA) or Merck (Darmstadt, Germany) and stored in stocks of 1 mg/ml in methanol (0.5% v/v diethylamine) at − 20 °C. Treatment solutions were prepared by evaporating the appropriate amount of LCB stock solution, dissolving it in DMSO by ultrasonication and diluting it for the required final concentration in respective growth media or in 10 mM MgCl_2_. Final treatment solutions were either 1 or 2% (v/v) of DMSO.

### Antifungal activity assays

For growth tests in liquid medium or on agar plates, those media optimal for the respective species were used. Potato Dextrose Agar (PDA; Carl Roth GmbH, Germany) or potato dextrose medium was used for *V. longisporum* and *F. graminearum*, and Vegetable Juice (VJ) medium^45^ for *S. sclerotiorum* and *S. indica.* For plate experiments, mycelial plugs of identical size were placed in the center of the plate, except for *V. longisporum,* which was inoculated with 10 μL of spore solution (containing 5000 spores) pipetted in the center of the plate. Growth of mycelia on the surface of the medium was documented over time and calculated as % growth relative to the total area of the plate. For determination of fungal biomass in liquid medium, mycelial plugs, or, in case of *V. longisporum*, 10 μL of spore solution were used to inoculate 20 mL of appropriate medium, which was supplemented with 1% DMSO (solvent control treatment) or different concentrations of t18:0 dissolved in DMSO (final DMSO concentration: 1%). Before reaching the stationary growth phase (5 d for *S. sclerotiorum* and *F. graminearum,* 10 and 12 days for *V. longisporum* and *S. indica*, respectively) on a shaker incubator at 22–24 °C in the dark, all fungal structures were recovered on filter paper by vacuum filtration. After 24 h of drying at 60 °C, fungal dry weight was calculated by subtracting the weight of the filter paper which was determined before.

### Antibacterial activity assays

To determine killing efficiency of t18:0, bacterial pellets from over night cultures were dissolved in 10 mM MgCl_2_ to an optical density of 0.002, and 300 μL of this solution was mixed with an equal volume of the treatment solution. Treatment solution was 10 mM MgCl_2_ containing either the respective LCB dissolved in a final concentration of 2% DMSO, or 2% DMSO (v/v) alone (control). Final OD was 0.001, and final concentrations of t18:0 were as indicated in Supplementary Fig. 2. 15–20 min after mixing bacteria with the treatment solution, the amount of surviving bacteria was determined by preparing serial tenfold dilutions of the bacteria, and plating these dilutions on Mueller–Hinton (MH) agar (Merck, Germany). Colony numbers were then determined after 24–48 h. Growth inhibition was calculated as the percentage of colonies developing relative to the control treatment. To photometrically determine *Pst* growth in liquid culture over time, bacteria were grown as described above and diluted in LB medium containing respective concentrations of t18:0 or t18:0-P dissolved in 2% DMSO, or in 2%DMSO (control) alone, to an OD of 0.01 in 96-well microtiter plates. Bacterial growth was measured in a microplate reader at a wavelength of 595 nm.

### Co-infiltration experiments

For plant co-infiltration experiments, bacteria were diluted in 10 mM MgCl_2_ to an OD of 0.01, as described in^13^. The bacterial solutions were infiltrated into leaves of *Arabidopsis thaliana* from the abaxial side and used for floating leaf discs cell death assays. Cell death assays were performed as described in^10^. In short, leaf discs were detached, washed by floating on ultrapure H_2_O, and five leaf discs per sample were equilibrated in 0.3 ml of ultrapure H_2_O. Conductivity of the solution was determined using a LAQUAtwin EC-11 conductivity meter (Horiba, Kyoto, Japan). After the last measurement, leaf discs in the treatment solution were heated in closed vials for 1 h at 100 °C and the conductivity representing 100% cell death was determined. Bacterial growth in the leaves was determined by quantitative real-time PCR, as described in^46^.

### Statistical analysis

Statistical tests were performed using the software package IBM SPSS Statistics (Ver. 26). Mycelial growth on plates over time (Fig. 1), bacterial growth over time (Fig. 3), and leaf cell death over time (Fig. 4b) were analyzed using univariate analysis of variance (ANOVA). Variance was calculated by defining as fixed factor the respective t18:0 (or t18:0-P) concentration(s) and the factor ‘time’ as a covariate. Statistical significance between more than two groups (multiple comparison) was validated by one-way ANOVA with a post-hoc Tukey HSD (Fig. 2).

## Supplementary Information

Supplementary Information.
