# Supplementary material for: The major plant sphingolipid long chain base phytosphingosine inhibits growth of bacterial and fungal plant pathogens
Source: Sci Rep. 2022 Jan 20;12:1081. doi: 10.1038/s41598-022-05083-4 (PMC8776846; doi:10.1038/s41598-022-05083-4)
Supplement: Supplementary file 1 — Supplementary Information. [file 41598_2022_5083_MOESM1_ESM.pdf]

Glenz et al.

Additional Material:

Supplementary Figures 1 to 3

Supplementary Figure 1

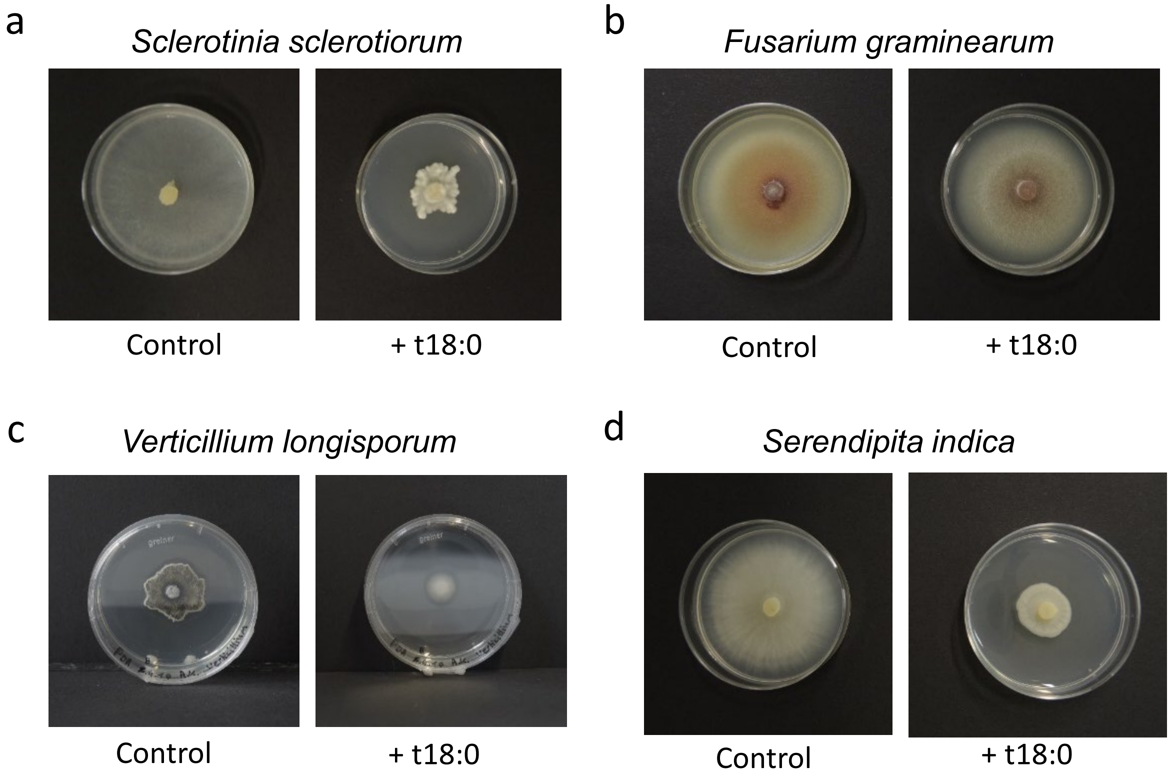

Supplementary Figure 1: Phytosphingosine inhibits growth of fungal hyphae on agar medium.

(a) *Sclerotinia sclerotiorum*, (b) *Fusarium graminearum*, (c) *Verticillium longisporum* and (d) *Serendipita indica*. Plates were inoculated in the center with a mycelial plug (a,b,d) or 10 μL of spore solution (containing 5000 spores) (c). Agar plates contained either 80 μM phytosphingosine (t18:0), or were prepared with the solvent (1% DMSO; Control). Photos show representative plates corresponding to data presented in Fig.1. Images were taken after 4 (a,b), 10 (c) or 11 days (d).

Supplementary Figure 2

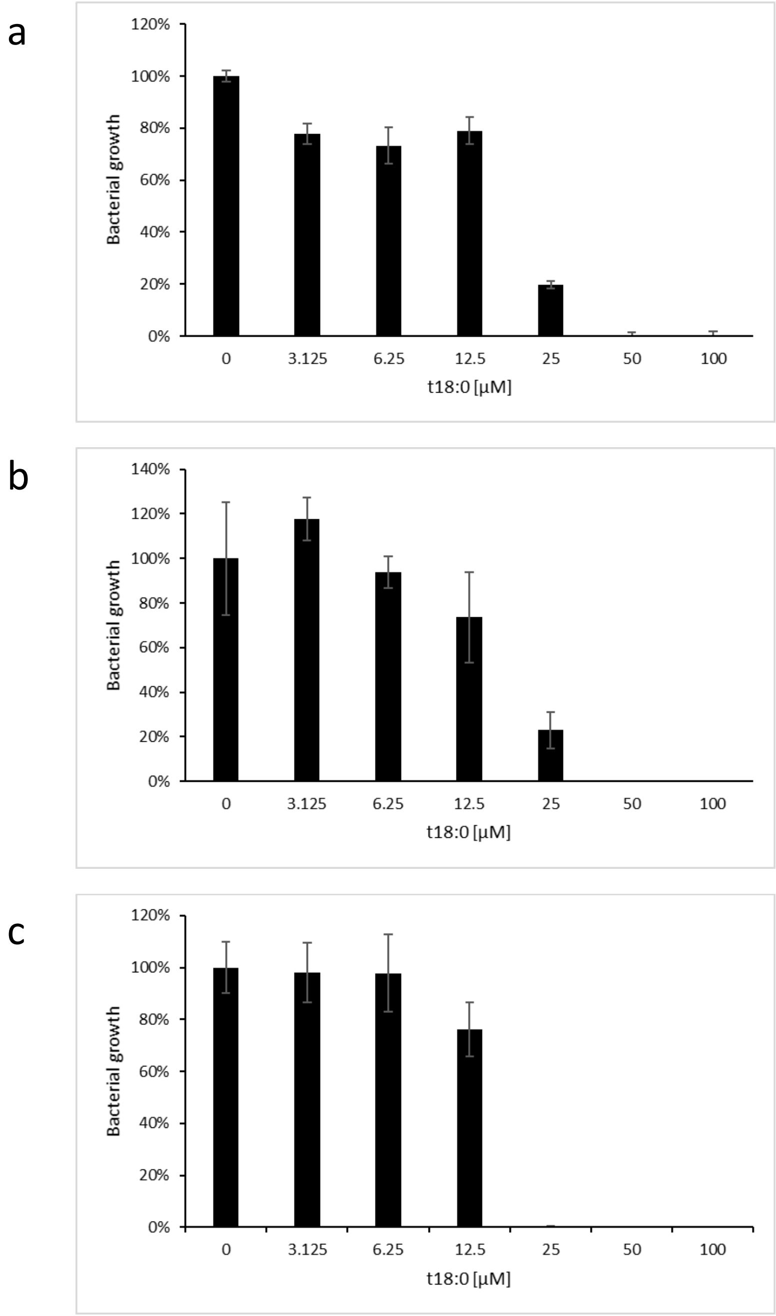

Supplementary Figure 2: t18:0 killing assay for three bacterial species

To determine killing efficiency of phytosphingosine for the three different bacterial species *Agrobacterium tumefaciens* (a), *Rhizobium radiobacter* (b) and *Pseudomonas syringae* (c), bacterial pellets were dissolved in 10 mM $MgCl_2$ and 300 mL of this solution was mixed with an equal volume of treatment solution, resulting in a final OD of 0.001. Treatment solutions were prepared with 10 mM $MgCl_2$, either containing the respective concentrations of t18:0 or solvent (2% DMSO). 15-20 min after mixing bacteria with the treatment solution, the amount of surviving bacteria was determined by plating serial 10-fold dilutions on Mueller-Hinton medium and determining colony numbers after 24-48 h. Killing efficiency was then calculated as the percentage of colonies developing relative to the solvent treatment, which was set to 100%. Indicated concentrations of t18:0 are final concentrations after mixing treatment solution with bacterial solutions. Values are means of four independent replicates for each treatment, with error bars indicating standard deviation. The experiment was repeated twice with similar results.

Supplementary Fig. 3

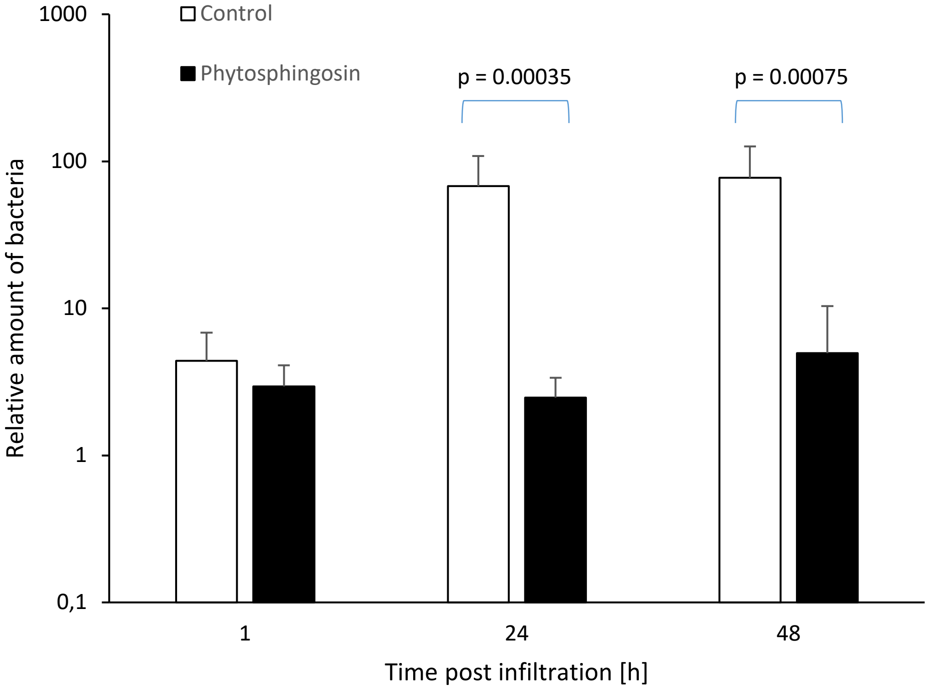

Supplementary Figure 3: Phytosphingosine inhibits growth of *Pseudomonas syringae* (*Pst*) in Arabidopsis leaves after co-infiltration with *Pst*

Pseudomonas syringae (*Pst*) was quantified in Arabidopsis leaves after co-infiltration of *Pst* with solvent (2% DMSO) alone (Control), or with 100 µM phytosphingosine (t18:0) dissolved in 2% DMSO. The amount of bacteria was determined relative to the amount of leaf material by extracting DNA at given time points and quantification of bacterial and plant DNA by quantitative real-time PCR, as described in (46). Relative amounts of bacteria were calculated as the ratio of Ct values obtained for the qPCR with primers specific for bacterial DNA to Ct values for qPCR with plant-specific primers. Values are means of nine independent replicates, with error bars indicating standard error. Results of pairwise Students T-Tests are indicated with brackets above respective treatments.

(46) Brouwer, M. *et al.* Quantification of disease progression of several microbial pathogens on Arabidopsis thaliana using real-time fluorescence PCR. *FEMS Microbiol Lett* **228**, 241-248, doi:10.1016/s0378-1097(03)00759-6 (2003).
